# Supplementary material for: What are the implications of using individual and combined sources of routinely collected data to identify and characterise incident site-specific cancers? a concordance and validation study using linked English electronic health records data
Source: BMJ Open. 2020 Aug 20;10(8):e037719. doi: 10.1136/bmjopen-2020-037719 (PMC7443310; doi:10.1136/bmjopen-2020-037719)

Supplementary appendix

Benefits and limitations of using individual and different combinations of linked English routine data sources in cancer epidemiology studies

Table 1: Number of patients identified with each cancer site using the gold standard algorithm

Cancer site	Number of patients
Oral Cavity (C00-06)	2097
Oesophageal (C15)	5212
Stomach (C16)	4016
Colorectal (C18-C20)*	22173
Liver (C22)	2230
Pancreas (C25)	5008
Lung (C34)	21978
Malignant melanoma (C43)	7282
Breast (C50)	29297
Cervix (C53)	1503
Uterus (C54-55)	4325
Ovaries (C56)	4157
Prostate (C61)	24888
Kidney (C64)	4086
Bladder (C67)	8871
Brain/CNS (C71-72)	2921
Thyroid (C73)	1314
NHL (C82-85)	6644
Multiple myeloma (C90)	2672
Leukemia (C91-95)	5279
Total	165953

Figure 1: Positive Predictive Value by age

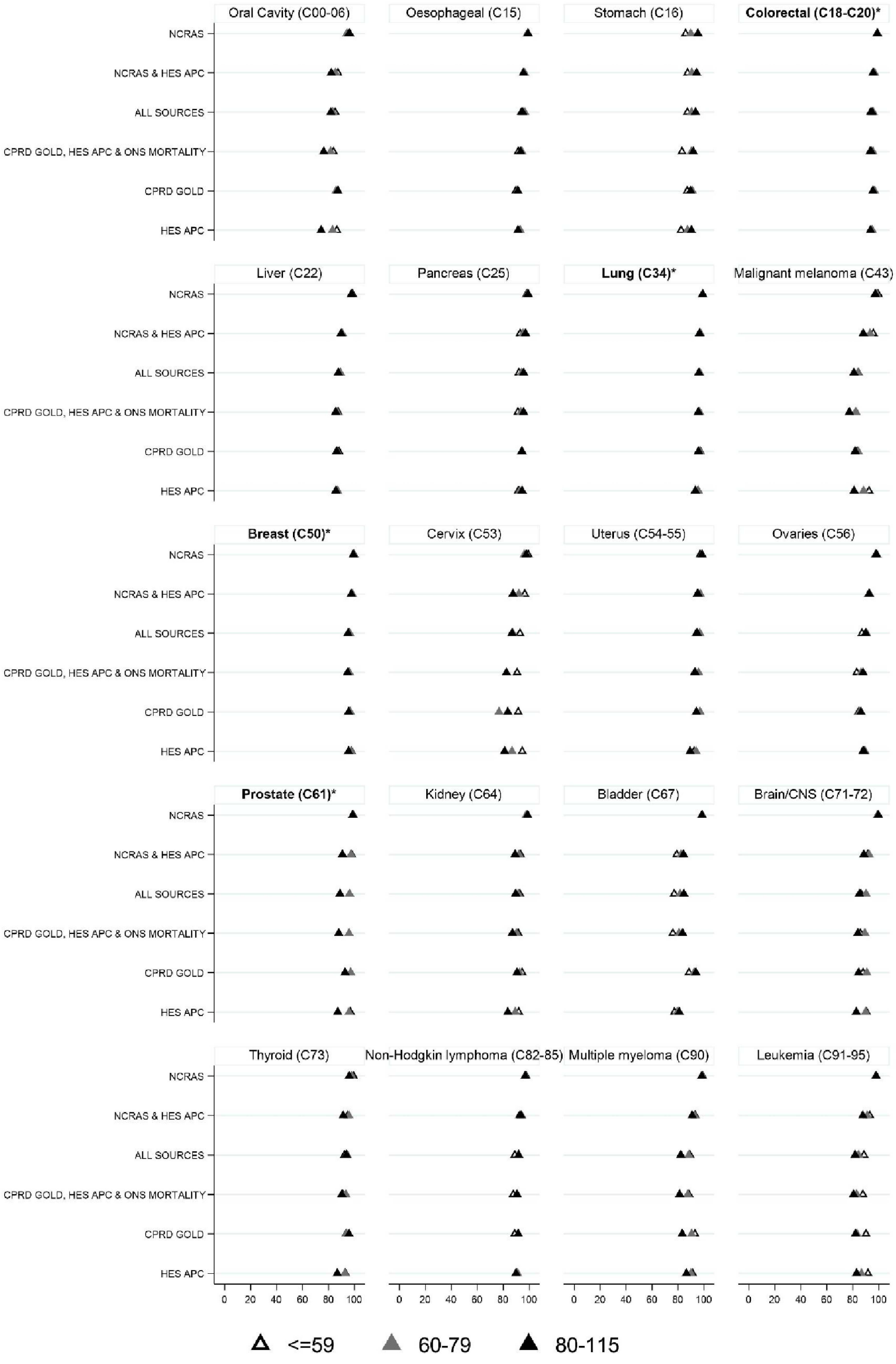

Figure 2: Positive Predictive Value by sex

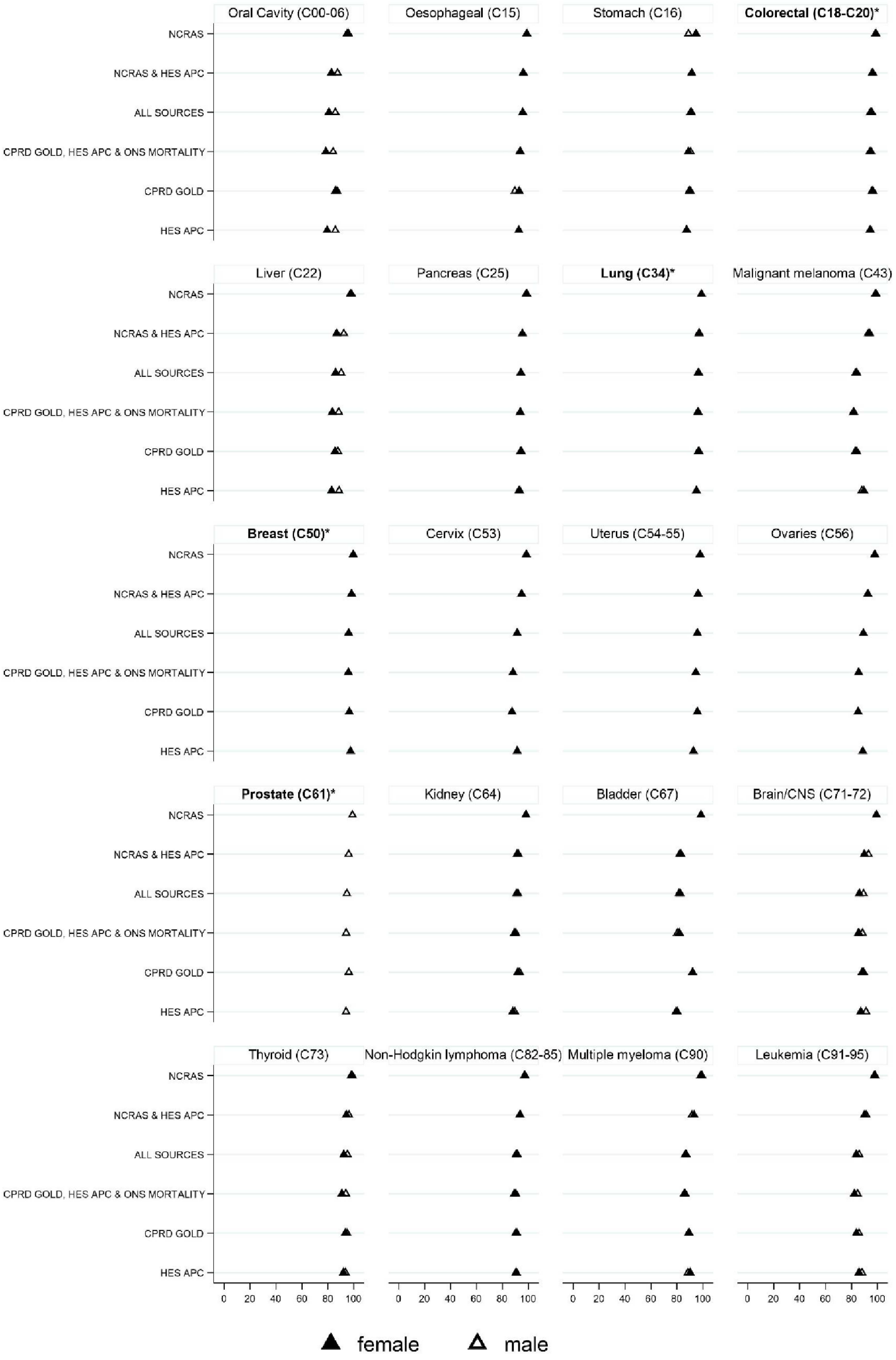

Figure 3: Positive Predictive Value by calendar year

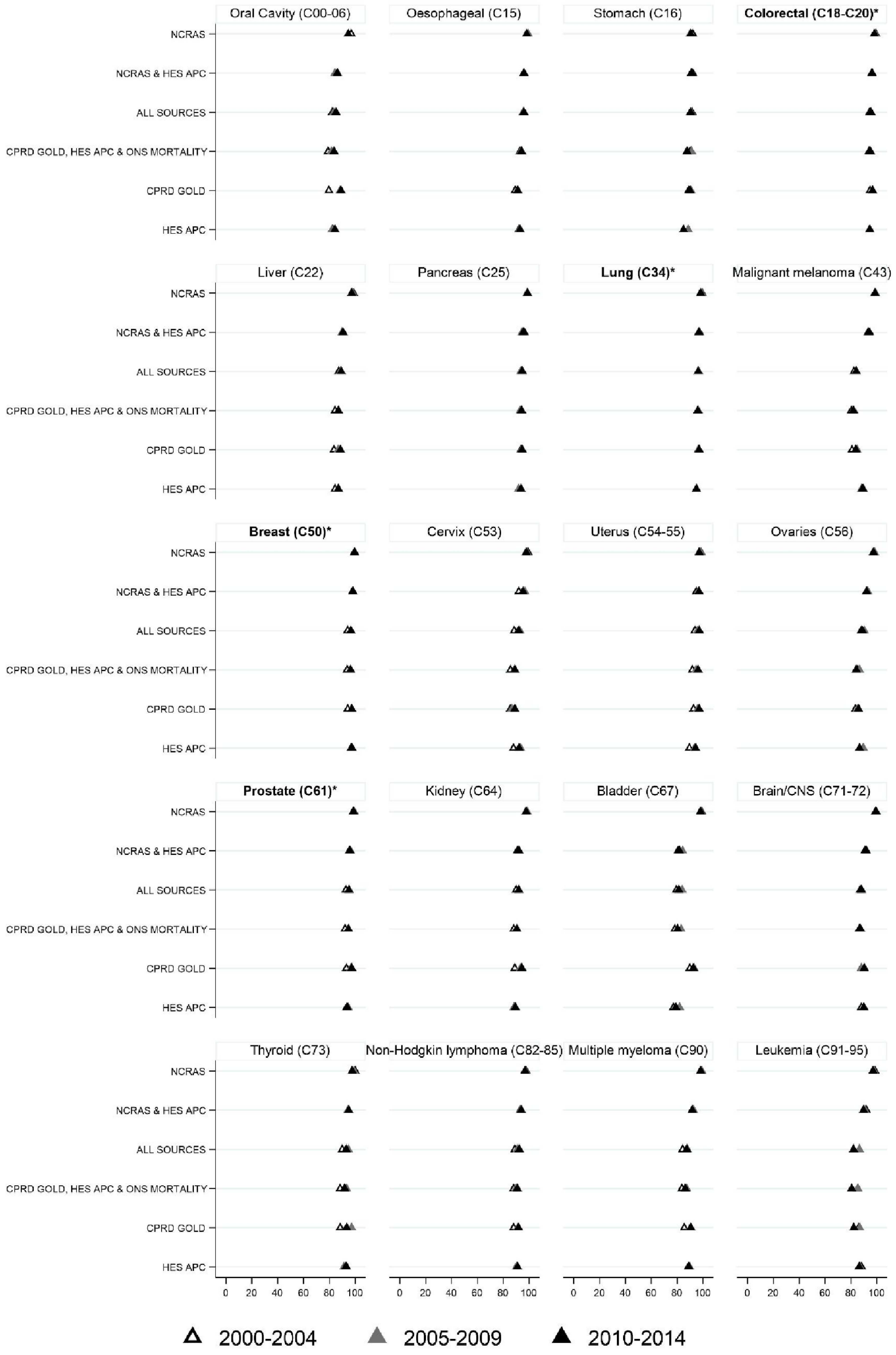

Figure 4: Sensitivity by age

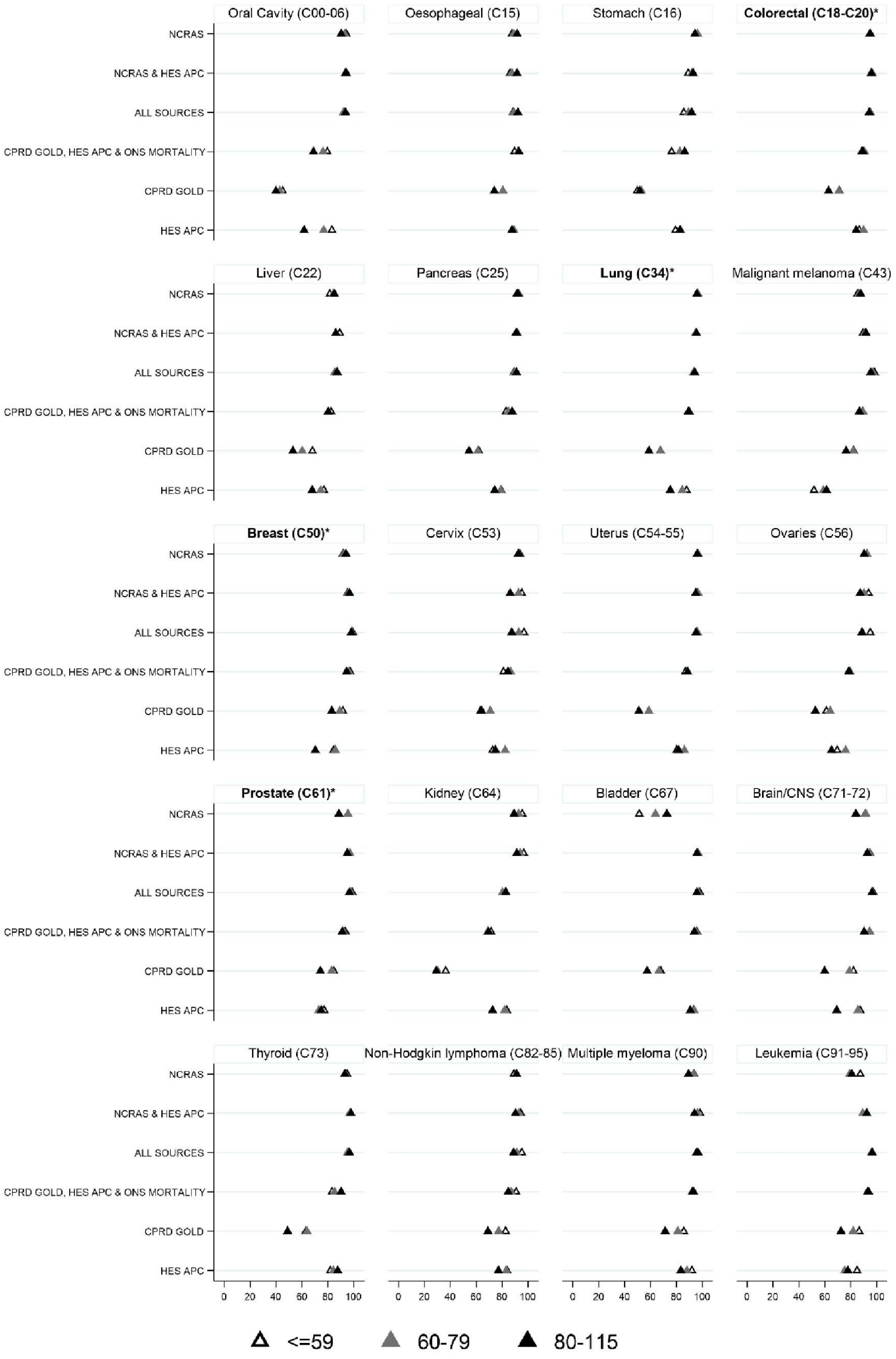

Figure 5: Sensitivity by sex

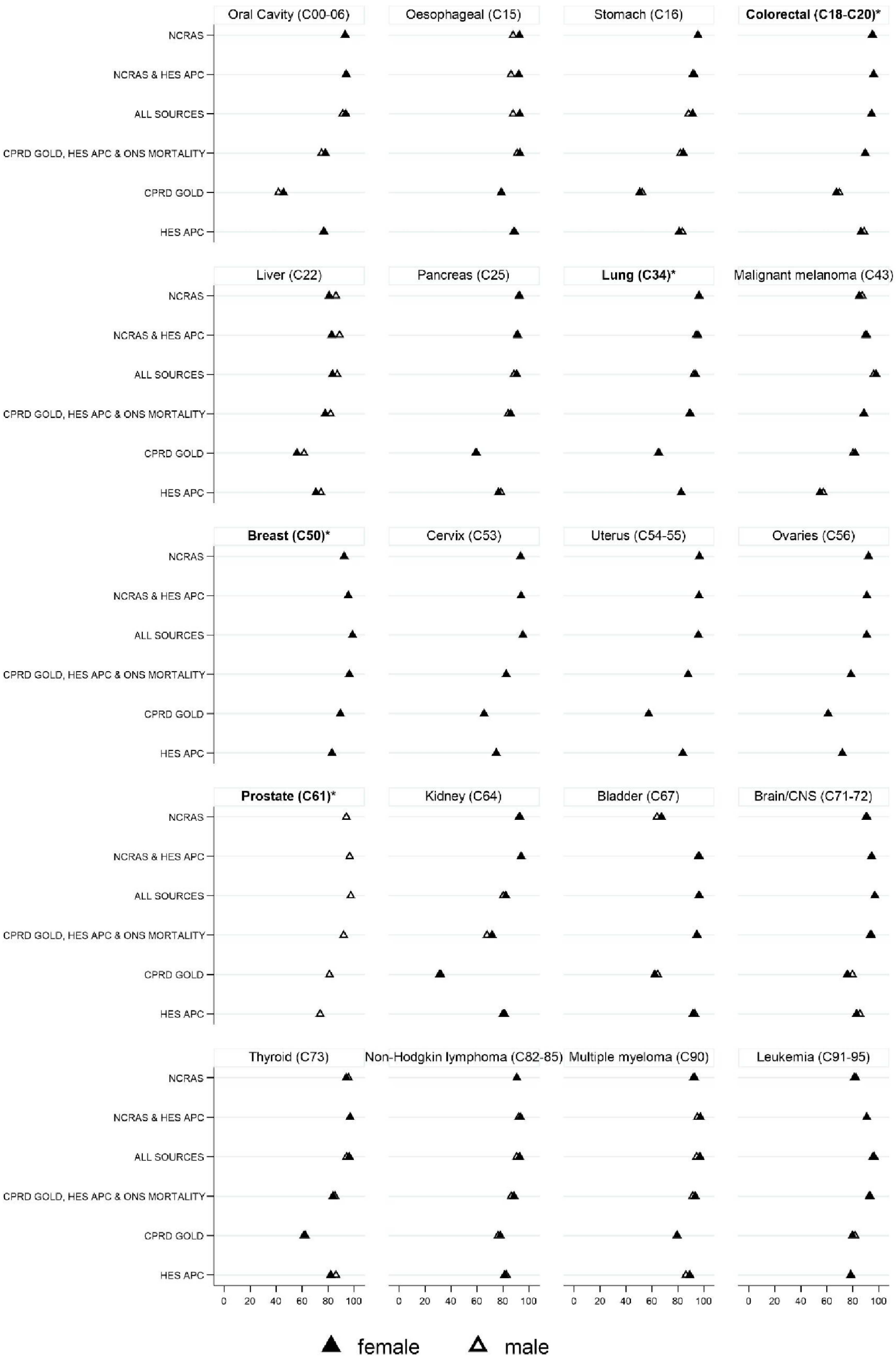

Figure 6: Sensitivity by calendar year

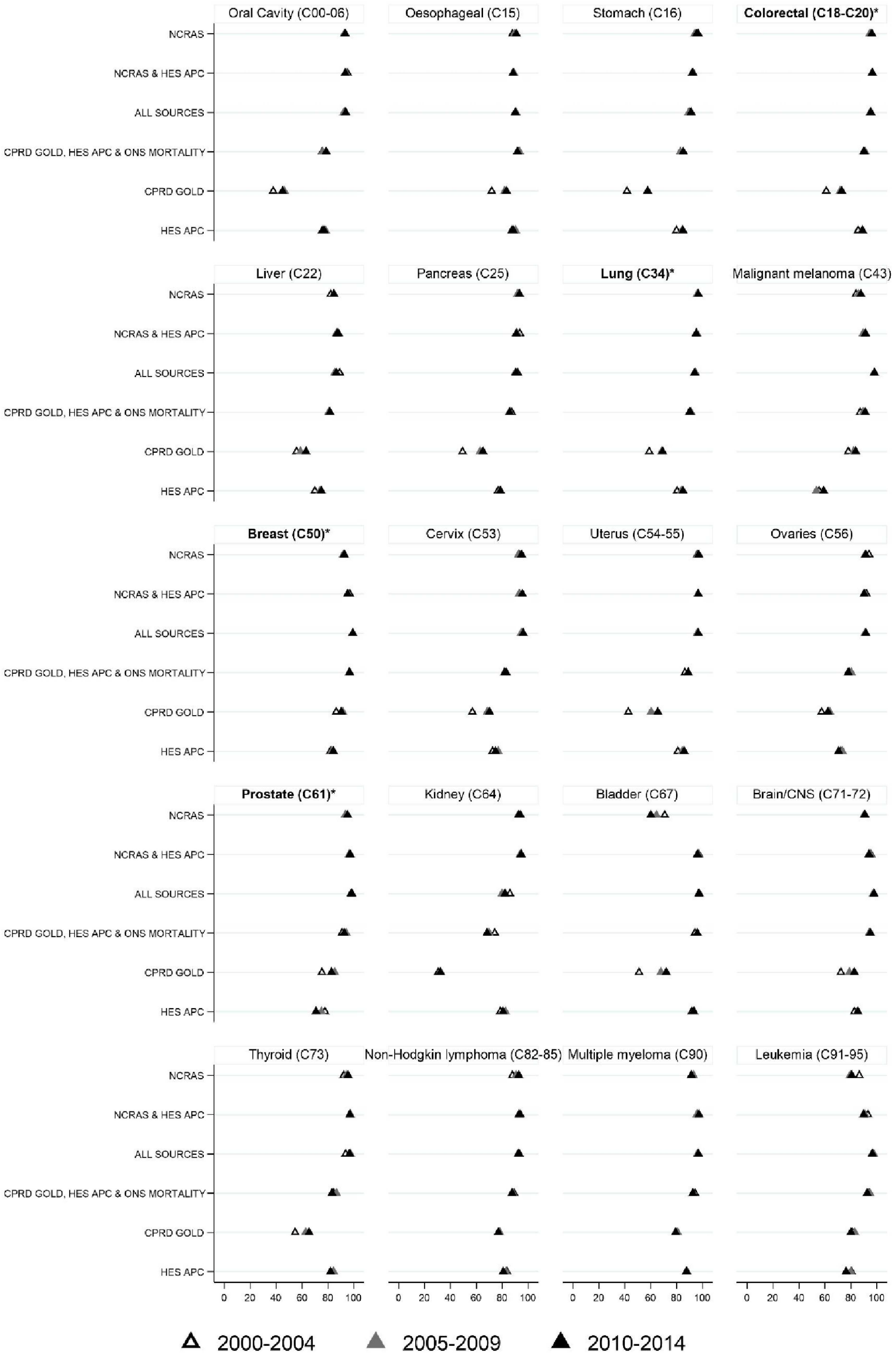

Figure 7: Output from logistic regression models with completeness of stage and grade as the dependent variables

Created using coefplot command in Stata http://repec.sowi.unibe.ch/stata/coefplot/

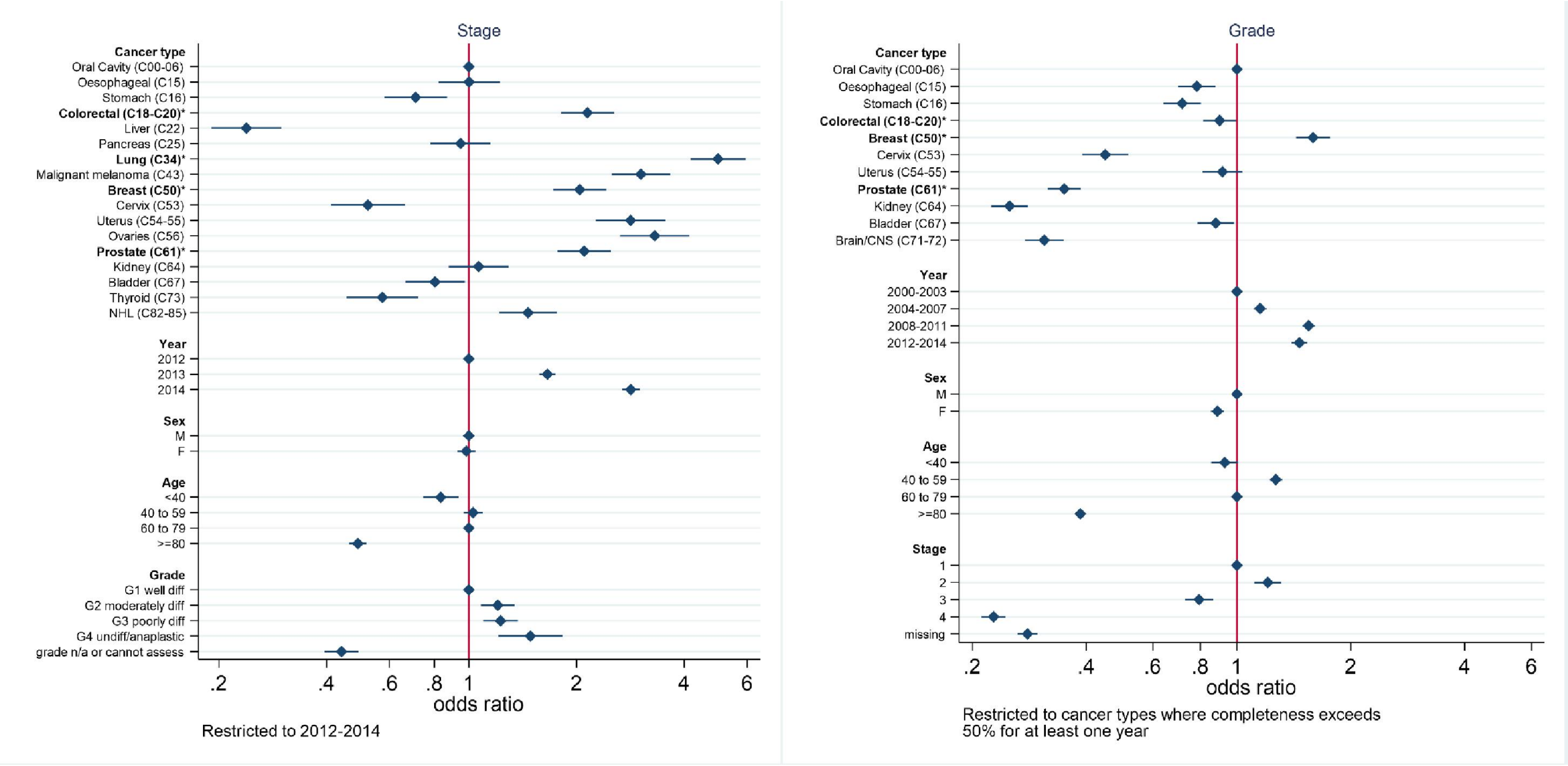

Figure 8: Recording of treatment modalities for patients identified using NCRAS data only

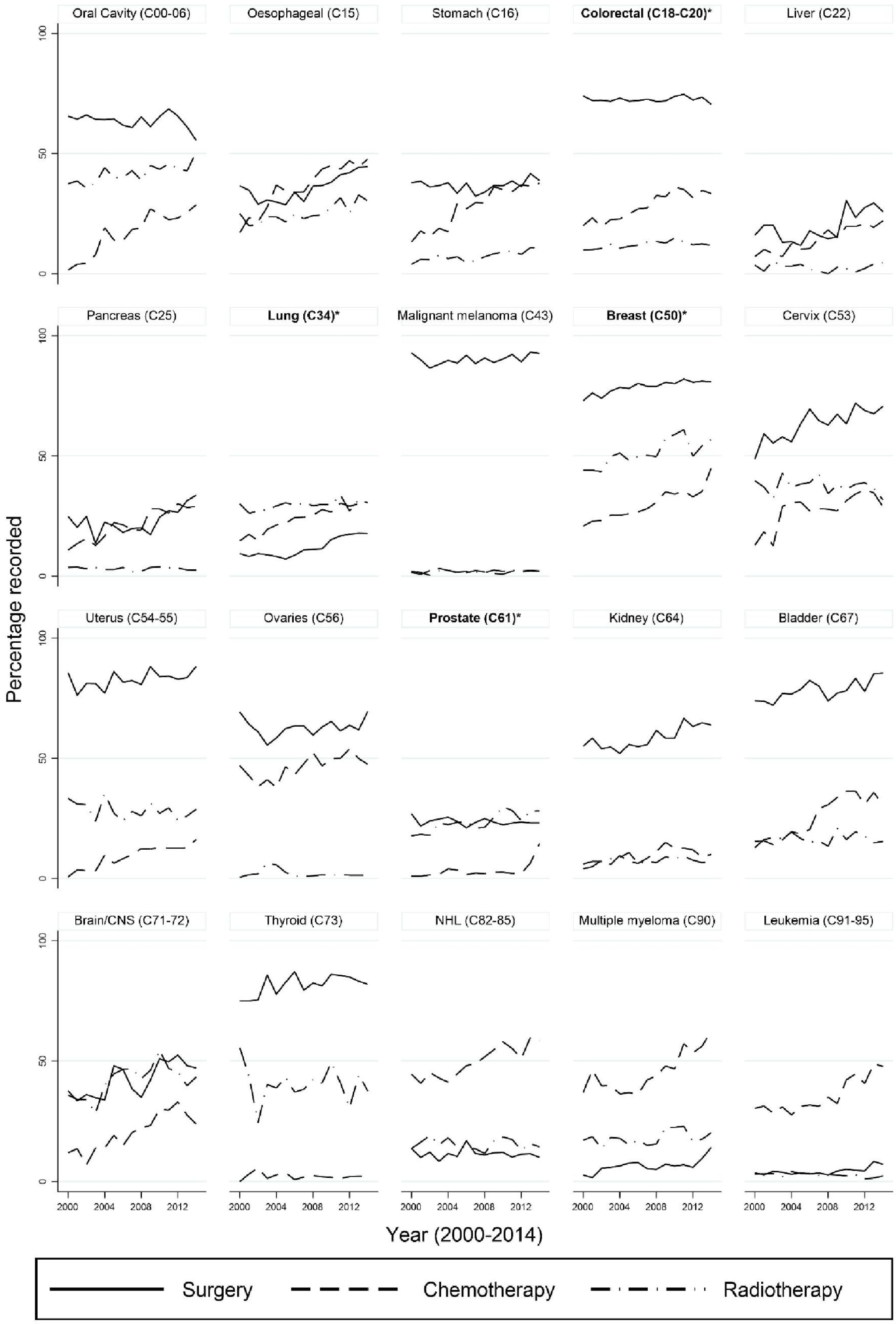

Supplement: Supplementary data [file bmjopen-2020-037719supp001.pdf]
